# A rapid colloidal gold immunochromatographic assay based on polyclonal antibodies against HtpsC protein for the detection of *Streptococcus suis*

**DOI:** 10.3389/fmicb.2023.1294368

**Published:** 2023-11-21

**Authors:** Yawei Lu, Sibo Wang, Xushen Cai, Min Cao, Qingyu Lu, Dan Hu, Qiong Chen, Xiaohui Xiong

**Affiliations:** ^1^College of Food Science and Light Industry, Nanjing Tech University, Nanjing, Jiangsu, China; ^2^Nanjing Bioengineering (Gene) Technology Center for Medicine, Nanjing, China

**Keywords:** *Streptococcus suis*, HtpsC, polyclonal antibody, immunochromatography, colloidal gold

## Abstract

An efficient and rapid immunochromatographic assay (ICA) has been engineered for the detection of *Streptococcus suis* (*S. suis*). The underpinning principle of this ICA test lies in the use of polyclonal antibodies (pAbs) decorated with colloidal gold, which are specific to *S. suis.* These pAbs were derived from rabbits immunized with type II histidine triad protein (HtpsC) and HtpsC-N of *S. suis*. The sensitivity of the ICA was noteworthy, identifying *S. suis* at bacterial concentrations as diminutive as 1.0 × 10^3^ CFU/mL. Moreover, the assay demonstrated respectable specificity and did not indicate false positives for other bacterial species (*Escherichia coli*, *Salmonella*, *Staphylococcus aureus*, *Listeria monocytogenes*, *Streptococcus pyogenes*, *Streptococcus lactis*, or *Enterococcus faecalis*). The assay was also capable of detecting multiple *S. suis* serotypes containing the *htpsC* gene, including serotypes 1–9, 12, 14, 16 and 23. Nonetheless, the detection of *S. suis* that lacks the *htpsC* gene remained beyond the capabilities of this assay. A simultaneous analysis of 16 samples utilizing PCR substantiated the reliability of the ICA test. The assay’s results can be procured within a 15-min window, making it a suitable option for field application. Broadly, this study underscores the potential of the HtpsC protein as a target antigen for the detection of *S. suis*, and proposes that the HtpsC protein be evaluated further in other detection assays specific for *S. suis*.

## 1 Introduction

*Streptococcus suis* (*S. suis*) is a significant zoonotic pathogen, transmitted to humans either through close contact with infected pigs or through the consumption of raw pork (Lun et al., 2007). Initial reports in 1954 identified meningitis, septicemia, and septic arthritis in piglets infected with *S. suis* (Wertheim et al., 2009), and the first human cases were diagnosed in Denmark in 1968 (Perch et al., 1968). Since then, *S. suis* infections have been reported in numerous countries spanning Western Europe, North and South America, and East and Southeast Asia, including Japan, China, and India (Mclendon et al., 1978; Lan et al., 2014; Dutkiewicz et al., 2017).

In recent decades, there has been a marked escalation in the global incidence of *S. suis* infection, with more than 1,500 cases documented across 34 countries between 2002 and 2013 (Dutkiewicz et al., 2017). Notably, Asia has seen prevalent cases of *S. suis* infections, particularly in China, Thailand, and Vietnam (Mai et al., 2008; Rayanakorn et al., 2018; Huang et al., 2019). China experienced two large-scale human *S. suis* infection outbreaks in 1998 and 2005, the latter involving over 200 infections (Du et al., 2017). Recurrences, such as the 2016 outbreak in Guangxi Province, are a significant concern (Mai et al., 2008; Rayanakorn et al., 2018). Without timely diagnosis and treatment, the mortality rate can reach up to 40% (Normile, 2005; Tang et al., 2006). Early symptoms, such as high fever, headache, and vomiting, mimic pyogenic meningitis caused by other bacteria, complicating early diagnosis and increasing patient risk (Suankratay et al., 2004). Hence, improved diagnostic techniques are urgently required.

Presently, *S. suis* infection diagnosis relies on pathogenic microbiological assays, molecular biology assays, and immunological assays (Arai et al., 2015; Zhang and Hao-ju, 2020). Conventional microbiology assays, however, suffer from being laborious and having low sensitivity (Xia et al., 2017). Recent advancements in molecular techniques have led to the development of new, more sensitive detection methods for *S. suis*, such as multiplex polymerase chain reaction (m-PCR) and fluorescence quantitative real-time-PCR (FQ-PCR) assays (Silva et al., 2006; Srinivasan et al., 2016; Denich et al., 2020). Yet, these methods depend heavily on skilled personnel and expensive equipment, limiting their widespread adoption (Xia et al., 2018). Conversely, immunochromatographic test (ICT) technologies offer a promising approach for efficient point-of-care testing, although existing assays have limited sensitivity and detect only a narrow spectrum of *S. suis*.

In 2011, a type II histidine triad protein (HtpsC) was first identified in the Chinese strain of *S. suis* 2 (Shao et al., 2011). The protein, shown to bind to components of the human extracellular matrix (ECM) complex, suggested that HtpsC is an adhesin and thus a potential detection target for *S. suis* (Li et al., 2015). Our previous work also found a similar three-dimensional structure shared by HtpsC and internalin A (InlA) from *Listeria monocytogenes* (Lu et al., 2021). Given the observation that HtpsC is a membrane surface protein, we hypothesized it could be exploited as a detection target in an assay for *S. suis*.

In this study, we developed a colloidal gold (CG) ICA using pAbs against HtpsC protein to detect *S. suis*. The method exhibits several beneficial characteristics, including short detection time, portability, high specificity, and naked-eye interpretability, making it suitable for practical and rapid field detection of *S. suis*.

## 2 Materials and methods

### 2.1 Bacterial strains and plasmids

All *S.suis* strains used in this study as depicted in Table 1 were kindly provided by Prof. Marecelo Gottschalk from Canada, Prof. Astrid de Greeff from the Greeff Laboratory in the Netherlands, and Prof. Ming Li from Army Medical University in China. The remaining strains were obtained from the Guangdong Microbial Culture Collection Center (GDMCC) and TransGen Biotech in China.

**Table 1:** Bacterial strains used in this study.

Strain	Laboratory name	Serotype	Reference/source	Culture medium	Growing temperature
*S.suis*	5,428	1	Prof. Marecelo Gottschalk	THB Broth	37°C	05ZYH33	2	Tang et al. (2006)	THB Broth	37°C	WZ48-1	2	Prof. Ming Li	THB Broth	37°C	T15	2	Prof. Astrid de Greeff	THB Broth	37°C	NCTC 10234	2	Prof. Marecelo Gottschalk	THB Broth	37°C	2,524	4	Prof. Marecelo Gottschalk	THB Broth	37°C	8,074	7	Prof. Marecelo Gottschalk	THB Broth	37°C	22,083	9	Prof. Marecelo Gottschalk	THB Broth	37°C	13,730	14	Prof. Marecelo Gottschalk	THB Broth	37°C
*E.coli*	GDMCC 801268		GDMCC	LB Broth	37°C
*L. monocytogenes*	ATCC 54004		GDMCC	Listeria Enrichment Broth Base	37°C
*E. faecalis*	ATCC 51299		GDMCC	Brain Heart Infusion Broth	37°C
*S. aureus*	ATCC 29213		GDMCC	LB Broth	37°C
*S. pyogenes*	ATCC 19615		GDMCC	Brain Heart Infusion Broth	37°C
*S. agalactiae*	GDMCC 1.408		GDMCC	Brain Heart Infusion Broth	37°C
*S. enteriditis*	ATCC 14028		GDMCC	LB Broth	37°C

### 2.2 Preparation of HtpsC-N recombinant protein

PCR was used to amplify the *htp*-domain of the *htpsC* gene sequence (designated *htpsC*-N) from *S. suis* strain 05ZYH33. The *htpsC* -N primers were:

F: ATGGGTCGCGGATCCGAATTCATGAACATACGACTTGTAGTG (*Eco*R I) R: TGGTGGTGCTCGAGTGCGGCCGCAATATAAGACTCCCACGG (*Not* I).

The *htpsC*-N gene fragment was cloned into the pET-28a linker region between its *Eco*R I and *Not* I site to obtain recombinant plasmid 28a-*htpC-*N. The HtpsC-N recombinant protein was expressed in *E. coli* BL21(DE3) and purified by Ni affinity chromatography. The kits used for cloning and affinity purification were ClonExpress^@^ II One Step Cloning Kit (Vazyme Biotech Co., Ltd., Nanjing, China) and Ni-NTA Agarose (QIAGEN., Germany) respectively and the protocols provided by the manufactures were followed. The recombinant protein was then identified by Western blot and indirect ELISA, respectively. *S. suis* infected pig sera and Peroxidase-conjugated goat anti-pig IgG (Biosynthesis Biotech Co., Ltd., Beijing, China) were used as primary and second antibodies, respectively, in both assays and results were shown in Supplementary Figure S1 and Supplementary Table S1.

### 2.3 Preparation of polyclonal antibodies (pAbs) against HtpsC-N recombinant protein

pAbs against HtpsC-N were prepared as follows. Two female, pathogen-free New Zealand white rabbits (6–8 weeks of age) were subcutaneously injected with 1 mL of PBS solution containing 300 μg recombinant protein, which had been previously emulsified with Freund’s complete adjuvant. Rabbits were boosted four times at two-week intervals with 150 μg of HtpsC-N recombinant protein emulsified in Freund’s incomplete adjuvant. Seven days after the final boost, blood samples were collected from the ear vein of each rabbit. Anti- HtpsC-N protein titers were determined by indirect ELISA (Supplementary Table S2). When antibody titers reached ≥1:128000, whole blood was collected from the carotid artery of each rabbit and, HtpsC-N antibodies were purified from sera by affinity chromatography and were identified by western blot using HtpsC-N antibodies as target antigen and anti-rabbit were purified from sera by affinity chromatography and were identified by western blot (Supplementary Figure S2).

### 2.4 Preparation of colloidal gold

Colloidal gold was produced by trisodium citrate reduction, using a previously described method with slight modifications (Na et al., 2020). All glassware was first washed with ultrapure water, cleaned using aqua regia, and finally washed with ultrapure water and dried before use. 100 mL of 0.01% gold chloride tetrahydrate (HAuCl_4_) solution was heated to boiling. 1% trisodium citrate solution was then added. To obtain colloidal gold of suitable particle size, the boiling solution was stirred continuously for 15 min until the color of the solution changed from dark to wine red. The solution was boiled for another 5 min and cooled to room temperature. The colloidal gold solution was characterized using an ultraviolet spectrophotometer (from 400 to 800 nm) and transmission electron microscopy (TEM).

### 2.5 Conjugation of colloidal gold with pAbs

The colloidal gold probe was prepared as previously described, with slight modifications (Xia et al., 2018). Anti-HtpsC pAbs obtained previously in our laboratory (Cai et al., 2022) were added into the colloidal gold solution, followed by continuous stirring for 30 min at room temperature. 1 mL of 1% PEG-20000 was added to the reaction system, and stirring continued for another 30 min. Then, 1 mL of 10% BSA was added and stirred for 30 min to block non-specific sites. The reaction solution was centrifuged at 12000 rpm for 20 min at 4°C. After centrifugation and removal of the supernatant, the precipitate was re-suspended in 1 mL dilution buffer (0.01 M Tris, 5% sucrose, 1% BSA, 1% PEG-20000, 10 μL Tween-20, pH = 8.0) and stored at 4°C.

### 2.6 Preparation of immunochromatographic strips

**Figure 1:**
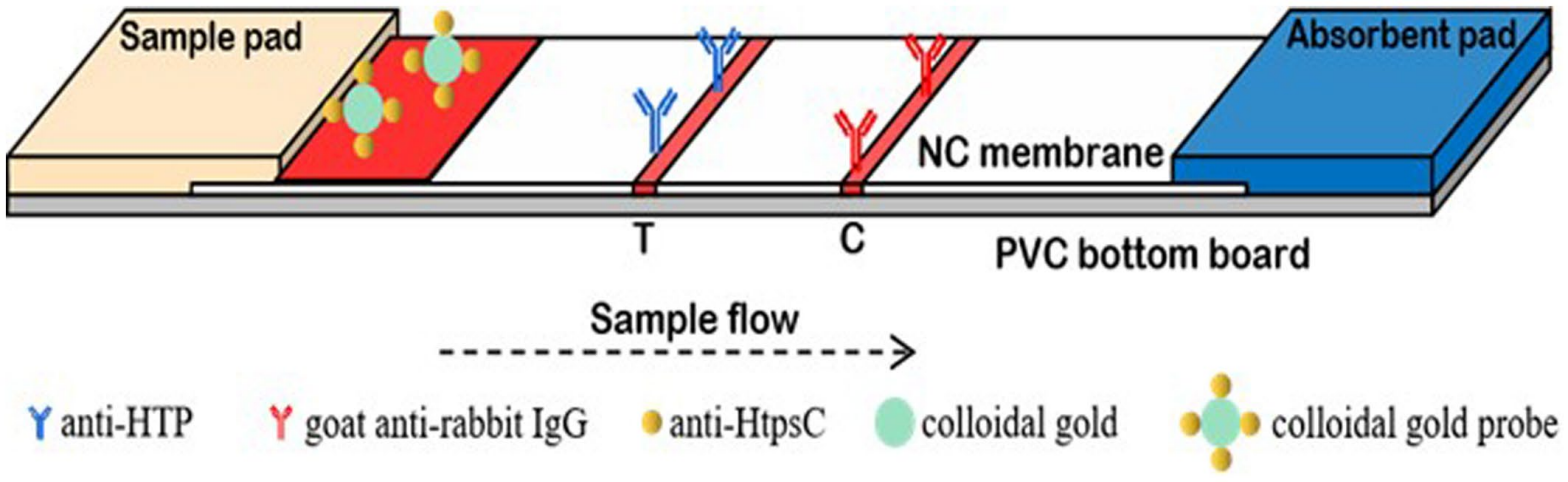
The structure of test strip. The anti-HtpsC-N polyclonal antibody and the goat anti-rabbit IgG were secured onto the nitrocellulose (NC) membrane, serving as the test (T) line and control (C) line, respectively. This setup was then utilized to assemble the colloidal gold immunochromatographic test strips.

Each 3 mm × 60 mm strip has five components: a polyvinyl chloride (PVC) support plate, a nitrocellulose (NC) membrane, an absorbent pad, and a conjugate pad (Figure 1). The NC membrane is glued to the middle of the PVC support plate. Using an Automatic Desktop Type Fine Spraying Machine (AUTOKUN, Hangzhou, China), HtpsC-N pAbs (1.0 mg/mL) are sprayed, at 1 μL /cm, onto the test area (“T”). Goat anti-rabbit IgG (1.0 mg/mL) is sprayed, at 1 μL /cm, onto the positive control area (“C”). These areas are situated 4 mm apart in the middle of the NC membrane. The membrane is dried for 4 h at 37°C then sealed in a plastic bag and stored in a desiccator at RT. A sample pad and absorption pad are each glued to one end of the PVC support plate, overlapping 2 mm onto the NC membrane. The conjugate pad is spotted with 5 μL/cm of colloidal gold conjugated HtpsC pAbs and dried at 37°C for another 2 h. The completed strips are stored at RT in a plastic cassette with silica desiccant gel.

### 2.7 Function of the CG-ICA detection system

In our assay, 100 μL of the sample solution is applied onto the sample pad. The solution then migrates across the nitrocellulose (NC) membrane toward the absorption pad due to capillary action. As the liquid passes through the test line (T line), bacteria in the sample solution that have been bound to Colloidal Gold-HtpsC polyclonal antibodies (CG-HtpsC pAbs) are immobilized by the HtpsC-N polyclonal antibodies (HtpsC-N pAbs) on the membrane. The liquid continues to migrate to the control line (C line), where the CG-HtpsC pAbs are immobilized by the goat anti-rabbit IgG. After about 15 min, the presence of red pigment at the T and C lines can be observed with the naked eye. As depicted in Figure 1, the presence of two red bands indicates a *S. suis* positive result. The C line, which serves as an assay control, must exhibit a red band regardless of whether the T line is red; if there is no red band at the C line, the test is considered invalid. The absence of a red band at the T line indicates a negative result. To evaluate the system, we tested each sample three times. This method provides quick and reliable results, making it a promising tool for *S. suis* detection, especially in resource-limited settings.

### 2.8 Optimization of experimental parameters

To achieve the best performance of the CG-ICT strips, we tested and optimized the following parameters: different volumes of 1% trisodium citrate (3, 2, 1.75, 1.5, 1.25 mL) used to generate colloidal gold particles; variable pH of the colloidal gold solution (5.0, 5.5, 6.0, 6.5, 7.0, 7.5, 8.0, 8.5 and 9.0), and different amounts of HtpsC pAbs (1, 2, 3, 4, 5, 6, 7, 8, 9, 10 μg) during conjugation with the colloidal gold probe.

### 2.9 Determination of the limit of detection (LOD) of CG-ICT strips

We used 100 μL of diluted *S. suis* 2 (from 10^8^ to 10^2^ CFU/mL) to evaluate the LOD of the strips.

### 2.10 Specificity of CG-ICT strips

Various bacterial strains were also tested to evaluate specificity, including different types of *S.suis* such as *S.suis* 15,428, *S.suis* 2 05ZYH33, *S.suis* 2 WZ48-1, *S.suis* 2 T15, *S.suis* 2 NCTC 10234, *S.suis* 42,524, *S.suis* 78,074, *S.suis* 922,083, *S.suis*14 13,730, *E.coli* GDMCC 801268, *Salmonella enteriditis* (*S. enteriditis*), *Staphylococcus aureus* (*S. aureus*), *Listeria monocytogenes* (*L. monocytogenes*), *Enterococcus faecalis* (*E. faecalis*), *Streptococcus pyogenes* (*S. pyogenes*), and *Streptococcus agalactiae* (*S. agalactiae*). Among these strains, *S. pyogenes*, *S. agalactiae* and *S. aureus* present relatively high genetic relationships with *S.suis.* A parallel analysis of these16 samples were also tested by PCR to evaluate the reliability of the ICA test.

### 2.11 Statistical analysis

Each experiment was performed with three replications. Data were analyzed by two-tailed, unpaired *t*-test. A *p* value of <0.05 was considered significant and a *p* value of <0.01 was considered highly significant.

## 3 Results and discussion

### 3.1 Multiple sequence alignment analysis of HtpsC-N

**Figure 2:**
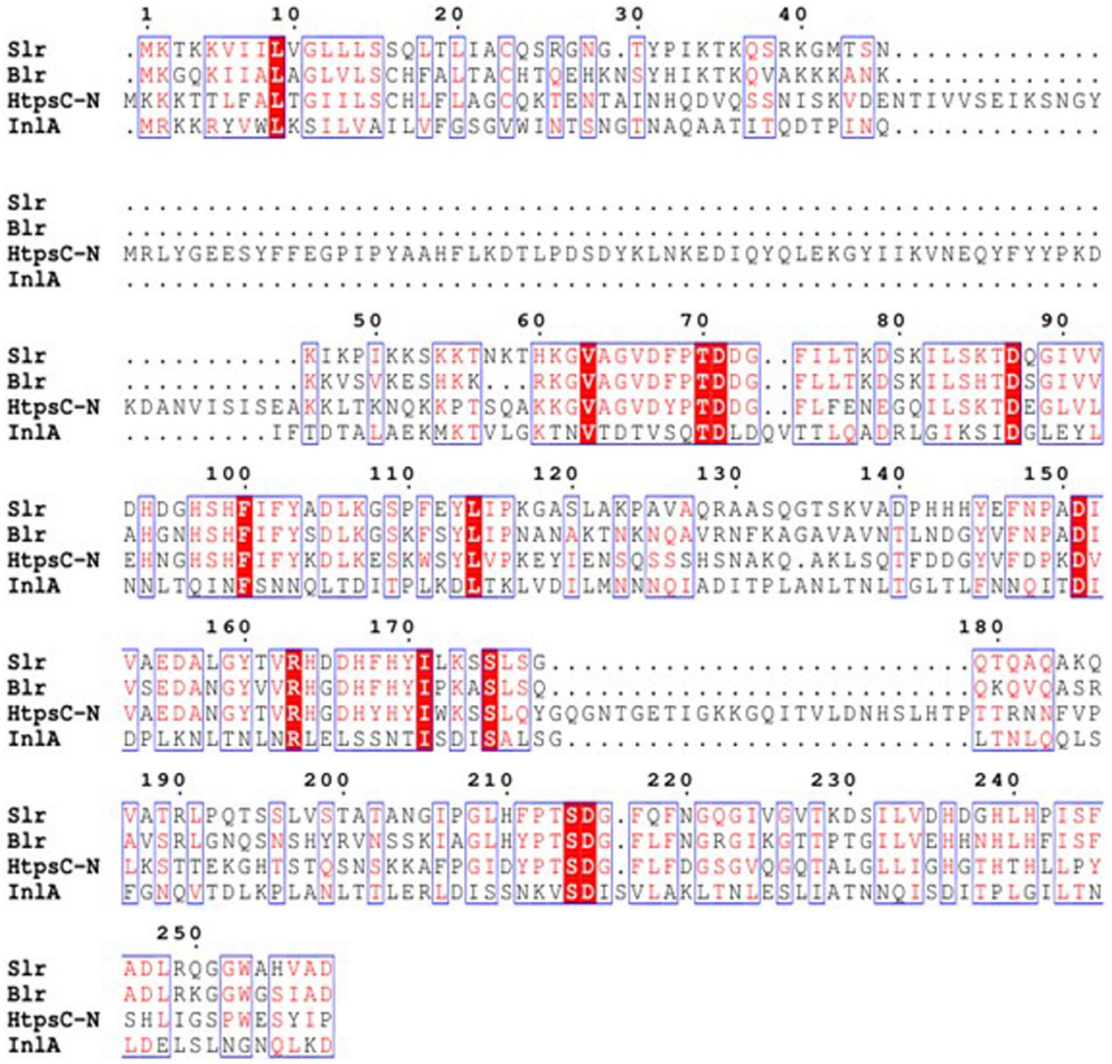
Multiple amino acid sequence alignments of *S. suis* HtpsC-N with homologous proteins Slr, Blr, and InlA. Slr is derived from *S. pyogenes* (Genbank accession: HQ908654.1), Blr from *S. agalactiae* (Genbank accession: DQ242614.1), and InlA from *L. monocytogenes* (GenBank accession: ABO32426.1). The ESPript 3.0 software was employed to execute the alignment.

Multiple sequence alignments employed by soft ESPript 3.0 have revealed that the N-terminal of the HtpsC protein (HtpsC-N) shares 13.8, 14.6, and 10.6% amino acid similarity with Streptolysin (Slr) of *S. pyogenes* (GenBank accession: HQ908654.1), Beta-lysin (Blr) of *S.agalactiae* (GenBank accession: DQ242614.1), and Internalin A (InlA) of *L. monocytogenes* (GenBank accession: ABO32426.1), respectively (Figure 2). Earlier, we reported that the full-length HtpsC protein shows 37.23, 37.07, and 27.32% amino acid similarity to the same three proteins (Lu, 2021). These findings suggest that HtpsC-N is more distinct compared to the full-length HtpsC protein, providing a greater discriminatory capability against these targets.

### 3.2 Optimization of experimental parameters

#### 3.2.1 Optimization of trisodium citrate volume

**Figure 3:**
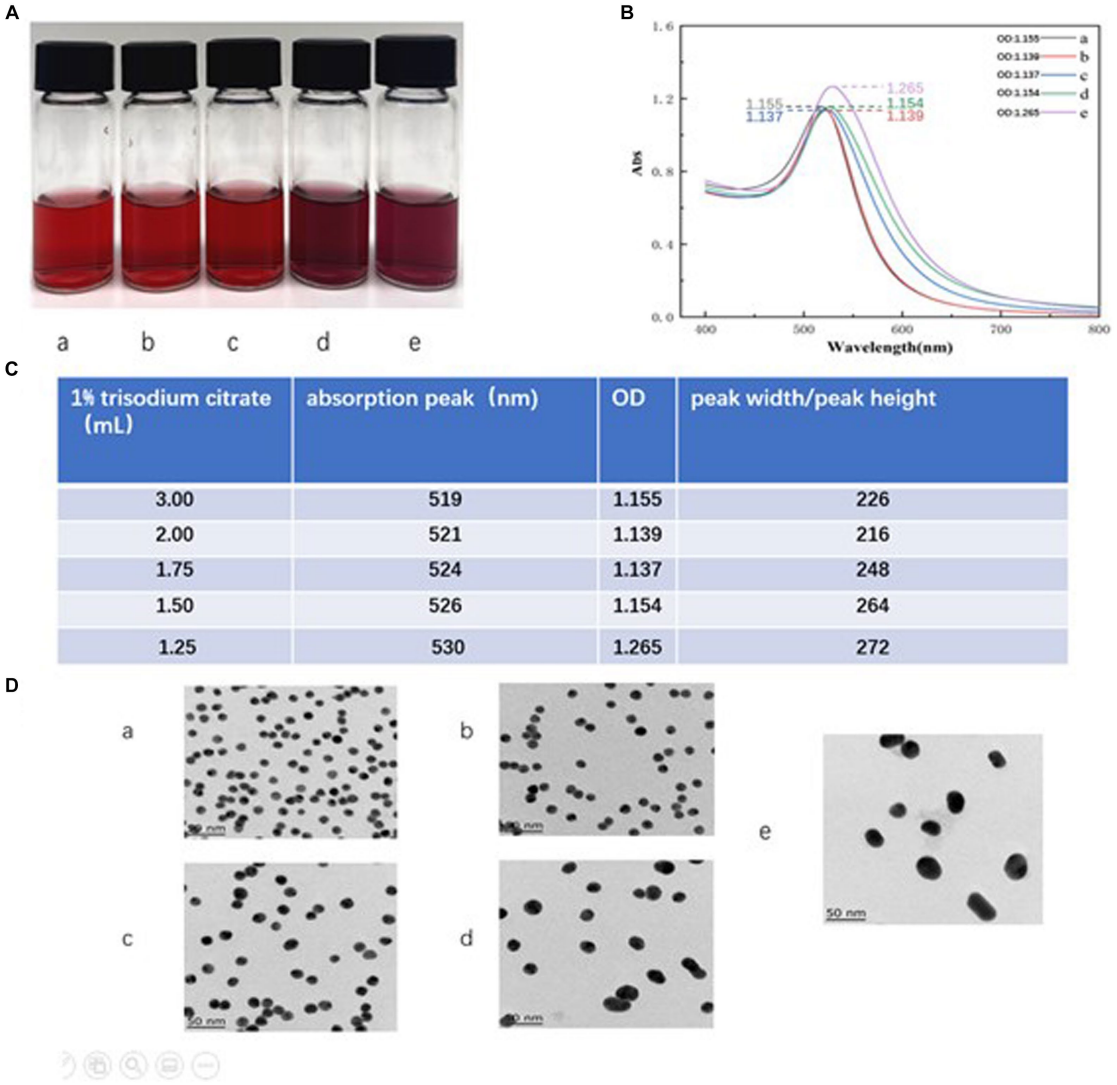
Optimization of trisodium citrate volume. **(A)** The color of the colloidal gold solution. Vials a-e represent the volumes of 1% sodium citrate added during colloid preparation, specifically, 3, 2, 1.75, 1.5, and 1.25 mL, respectively. **(B)** The absorption peaks scanned by UV full wavelength scanner. The plots a-e correspond to the waveforms of the colloid solutions when the volumes of 1% sodium citrate added were 3, 2, 1.75, 1.5, and 1.25 mL, respectively. **(C)** Showcases calculations derived from the scans in **(B)**, formatted as a table. The labels a–e indicate the absorption peaks and the ratio of peak width to peak height when the volumes of 1% sodium citrate added were 3, 2, 1.75, 1.5, and 1.25 mL, respectively. **(D)** TEM images of colloidal gold solution. The labels a–e represent the TEM images of solutions from vials a–e when volumes of 3, 2, 1.75, 1.5, and 1.25 mL of 1% sodium citrate were added, respectively.

The size and uniformity of the colloidal gold particles are known to affect the sensitivity of the assay. Therefore, optimization of the amount of trisodium citrate used in the colloid preparation is crucial. In our experiment, we observed that the colloidal gold solution exhibited the expected bright red coloration when 3 mL, 2 mL, and 1.75 mL of 1% trisodium citrate were used, as shown in Figure 3A (a–c). However, when 1.5 mL and 1.25 mL of 1% trisodium citrate were used, an abnormal purple or red-purple coloration was observed (Figure 3A, d–e).

Particle size distribution of colloidal gold can be preliminarily assessed using UV full wavelength scanning. A higher absorption peak indicates a larger particle size, while a smaller peak width-to-peak height ratio indicates better size uniformity (Frens, 1973). Our results, shown in Figure 3B (a–e), suggest that as the amount of 1% sodium citrate decreases (from 3, 2, 1.75, 1.5, to 1.25 mL), the absorption peak gradually shifts to the right, indicating a gradual increase in particle size. The solution with 2 mL of 1% sodium citrate showed the minimum peak width-to-peak height ratio, suggesting maximum particle size uniformity within the colloid (Figure 3C).

Comparison of transmission electron microscope (TEM) images revealed that the colloidal gold particles obtained using 2 mL and 3 mL of 1% sodium citrate (vials a and b) displayed better uniformity and regular spherical profiles. However, particles from vials c, d, and e (using 1.75 mL, 1.5 mL, and 1.25 mL of trisodium citrate, respectively) exhibited more irregular shapes. Thus, based on these results, we concluded that 2 mL of 1% sodium citrate is the optimal amount for generating uniform colloidal gold particles. The average particle size, as measured using the Image J application, was 15 nm (Figure 3D, b).

#### 3.2.2 Optimization of pH for preparation of colloidal gold solution

**Figure 4:**
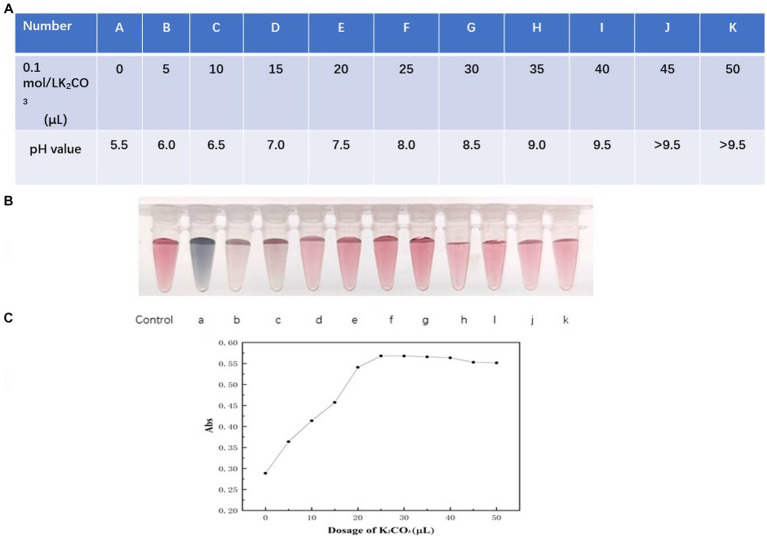
Optimization of pH of colloidal solution. **(A)** The pH value of the colloidal solution was adjusted by adding varying volumes of 0.1 mol/L K_2_CO_3_, presented as a table format. The data is presented in a tabular format wherein A–K indicate pH values from 5.5 to >9.5 corresponding to the varying volumes of 0.1 mol/L K_2_CO_3_ added. **(B)** The color variations of colloidal gold solution. Vials labeled a–k represent the color of the colloidal gold solution when the pH values were 5.5, 6.0, 7.0, 7.5, 8.0, 8.5, 9.0, 9.5, and > 9.5, respectively. **(C)** Absorption at 521 nm of colloidal solution relating to various dosage of K_2_CO_3._ The x-axis represents the volumes of 5, 10, 15, 20, 25, 30, 35, 40, 45, 50 μL of 0.1 mol/L K_2_CO_3_ respectively, corresponding to pH values from 5.5 to 9.5. The y-axis presents the absorption measured at 521 nm.

The formation of the colored compound primarily depends on the electrostatic attraction between the colloidal gold particles and the HtpsC polyclonal antibodies (pAbs). The pH of the buffer significantly influences the uniformity and quantity of the compound formed (Yang, et al., 2010; Yang et al., 2022). A lower pH fosters precipitation, while higher pH values decrease the amount of labeled product. We adjusted the pH value of the colloidal solution by adding varying volumes of 0.1 mol/L K_2_CO_3_, as presented in Figure 4A (table format).

As demonstrated in Figure 4B (vials a–d), when the pH is less than 7.5, precipitation occurs, causing the products to adhere to the vial walls. This is observable to the naked eye, and the solution takes on a blackish or purple color. However, when the pH is 7.5, 8, or 8.5, the color of the solution (Figure 4B, vials e–g) closely matches the reference colloidal gold solution. As shown in Figure 4C, absorption at 521 nm increased as the volume of 0.1 mol/L K_2_CO_3_ rose from 0 to 25 μL (corresponding to a pH shift from 5.5 to 8.0), reaching a maximum at 25 μL (pH = 8.0) and remaining stable beyond this point.

At a pH of 8.0, the environment is near the isoelectric point of HtpsC pAbs. At this pH, the force between HtpsC pAbs and colloidal gold is maximized (Sun et al., 2014), which facilitates the formation of a stable gold-labeled antibody solution. Therefore, considering these results, we conclude that the optimal pH for preparing gold-labeled antibodies is 8.0.

#### 3.2.3 Optimization of HtpsC pAbs content

**Figure 5:**
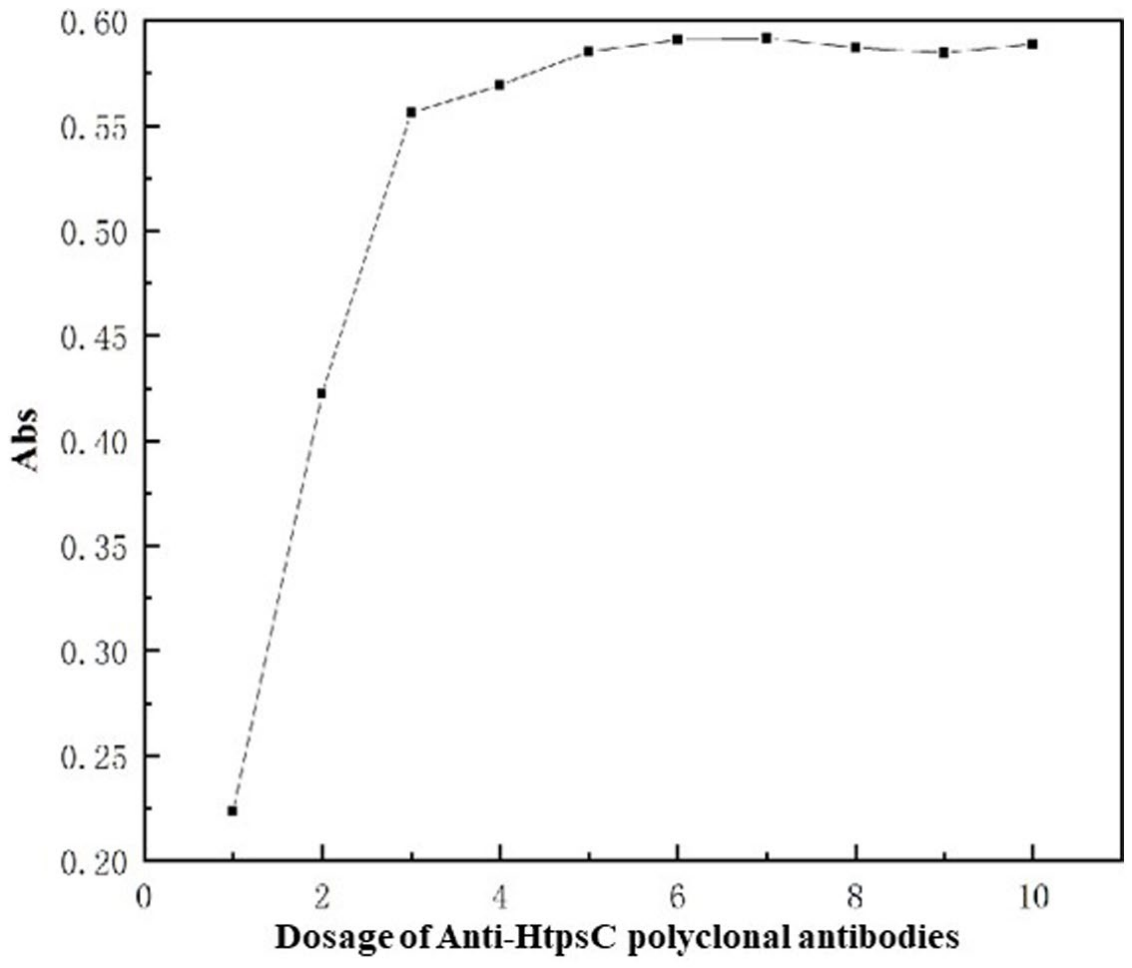
Optimization of amount of HtpsC pAbs used for conjugation. The x-axis represents the varying amounts of HtpsC pAbs used, specifically, 1, 2, 3, 4, 5, 6, 7, 8, 9, and 10 μg, respectively. The y-axis illustrates the absorption, measured at 521 nm.

We added varying amounts of HtpsC polyclonal antibodies (pAbs) to the conjugation reaction with 1 mL of colloidal gold particles at the optimal pH value of 8.0. As demonstrated in Figure 5, the absorption at 521 nm increased as the quantity of HtpsC pAbs escalated from 1 to 6 μg. The absorption peaked at 6 μg and remained stable beyond this point (7–10 μg).

Given these findings, we determined that 6.6 μg is the optimal quantity of HtpsC pAbs to be used for labeling in a reaction that contains 1 mL of colloidal gold particles. This amount ensures the maximum absorption at 521 nm, indicating optimal conjugation of the antibodies with the colloidal gold particles.

### 3.3 LOD determination of CG-ICT strips

**Figure 6:**
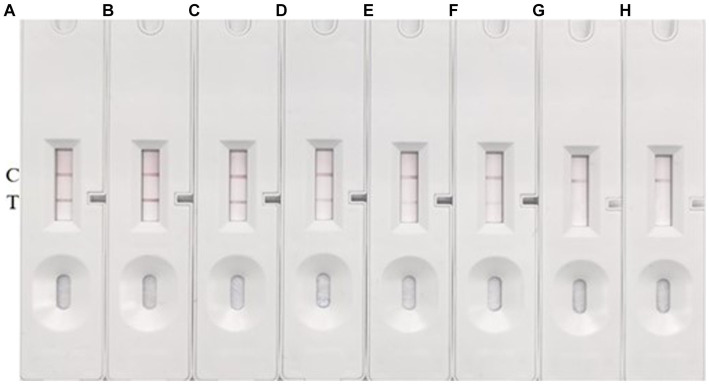
LOD determination. **(A–G)** Represent the concentrations of *S. suis* 2 in the amounts of 1 × 10^8^ CFU/mL, 1 × 10^7^ CFU/mL, 1 × 10^6^ CFU/mL, 1 × 10^5^ CFU/mL, 1 × 10^4^ CFU/mL, 1 × 10^3^ CFU/mL, and 1 × 10^2^ CFU/mL, respectively. **(H)** Represents a blank control using THB. The presence of two red bands at the *T* (test) and C (control) lines signifies a positive result for *S. suis.* Conversely, the absence of a red band at the T line indicates a negative result.

To establish the limit of detection (LOD) of our method, we cultured *S. suis* 2 (05ZYH33) to a concentration of 1 × 10^8^ CFU/mL, followed by serial dilution in 10-fold steps ranging from 10^8^ to 10^2^ CFU/mL. We then used these diluted bacterial suspensions as the antigen, with sterile Todd Hewitt Broth (THB) medium serving as a negative control. The results are documented in Figure 6. The test line (T line) was clearly red at concentrations of *S. suis* 2 (05ZYH33) higher than 1 × 10^5^ CFU/mL. As anticipated, the intensity of the T line color decreased as the concentration of *S. suis* 2 (05ZYH33) decreased. However, it remained faintly visible at a concentration of 1 × 10^3^ CFU/mL. No T line was visible at a concentration of 1 × 10^2^ CFU/mL. Multiple replicate experiments consistently showed that both the control (C) and test (T) lines were visible at a concentration of 1 × 10^3^ CFU/mL. No background signal was observed on the test strip under any of the tested conditions.

Based on these results, we conclude that the LOD of our method under these conditions is 1 × 10^3^ CFU/mL. This means that our method can reliably detect *S. suis* 2 (05ZYH33) at concentrations as low as 1 × 10^3^ CFU/mL.

### 3.4 Specificity of CG-ICT strips

**Figure 7:**
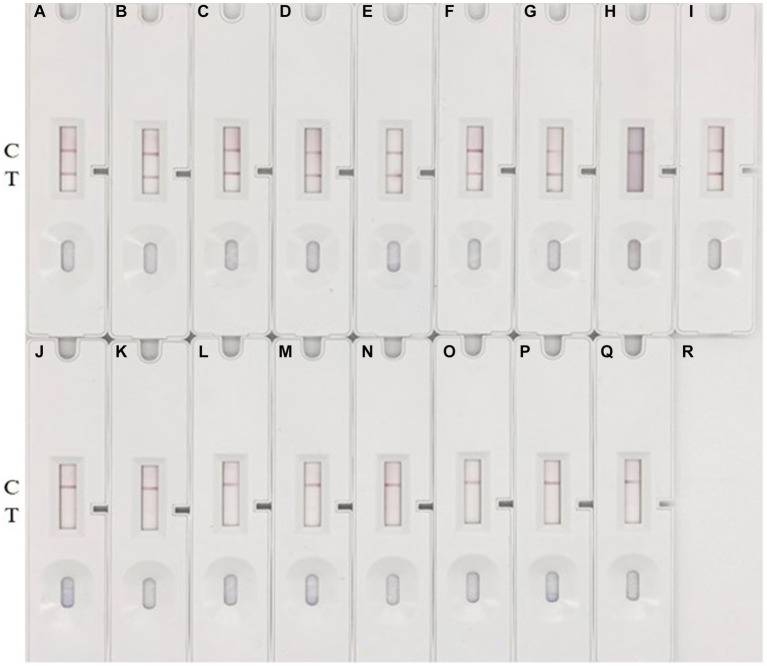
Specificity test. **(A–Q)** represent various bacteria: **(A)** *S. suis* 2 (05ZYH33), **(B)** *S. suis* 2 (T15), **(C)** *S. suis* 2 (WZ48-1), **(D)** *S. suis* 2 (NCTC10234), **(E)** *S. suis* 15,428, **(F)** *S. suis* 42,524, **(G)** *S. suis* 78,074, **(H)** *S. suis* 9, 22,083, **(I)** *S. suis* 1,413,730; **(J)** *E. coli* GDMCC 801268; **(K)** *S. enteriditis* ATCC 14028, **(L)** *S. aureus* ATCC 29213, **(M)** *L. monocytogenes* ATCC 54004, **(N)** *S. pyogenes* ATCC 19615, **(O)** *S. pyogenes* ATCC 19615, **(P)** *E. faecalis* ATCC 51299, and **(Q)** blank control with THB medium. The appearance of two red bands at the *T* (test) and C (control) lines signifies a positive result for *S. suis*. Conversely, the absence of a red band at the T line indicates a negative result.

To evaluate the specificity of the method, seven common pathogenic bacteria (*E. coli*, *S. enteriditis*, *S. aureus*, *L. monocytogenes*, *S. pyogenes*, *S. agalactiae*, *E. faecalis*) and five different serotypes of *S. suis* were selected and cultured to 1 × 10^6^ CFU/mL, and then culture samples were loaded onto the prepared colloidal gold immunochromatographic test strips. Representative results are shown in Figure 7. In multiple replicate experiments, the method demonstrated good specificity. None of the seven common pathogenic bacteria yielded positive signals. Among the six different serotypes of *S. suis*, all were positive except for type 9. The experimental results suggest that the method can detect multiple serotypes of *S. suis* containing the HtpsC protein gene. However, it cannot detect *S. suis* serotypes that do not express the HtpsC protein. A parallel analysis of these 16 samples by PCR confirmed the reliability of the immunochromatographic assay (ICA) test (refer to Supplementary Figure S3).

While *S.suis* is known to have 29 serotypes and some untypable strains, due to limited resources, we only tested 9 strains from 6 serotypes. To better understand the distribution of the *htpsC* gene among *S.suis* strains, we used the GenBank databases, SnapGene software (Version 6.0.2), and referenced primers for species and serotypes (Liu et al., 2013; Okura et al., 2014).

Our search yielded a total of 151 whole genomic sequences of *S. suis* strains. These strains represent various serotypes (1–10, 12, 16, 23, 24, 29, 30, 31, and a minority of non-serotypeable strains) (see Supplementary Table S3). It’s noteworthy that there might be strains of other serotypes that have not been sequenced yet. The *htpsC* gene was found in 119 out of 151 strains (78.8%), with a typical similarity of 99%. No *htpsC* gene was found in the remaining 32 strains. The 119 strains containing the *htpsC* gene were serotyped as 1–9, 12, 14, 16, 23, and a non-serotypeable strain. The 32 strains lacking the *htpsC* gene were serotyped as 4, 5, 10, 16, 24, 29–31, 33, and 9 non-serotypeable strains.

Our results suggest that most *S. suis* strains across various serotypes likely contain the *htpsC* gene. Interestingly, strains of the same serotype can either contain or lack the *htpsC* gene, as observed in serotypes 4, 5, and 16. This finding aligns with our experimental results using *S. suis* 22,083, a serotype 9 isolate provided by Marcelo Gottschalk from Canada. Contrary to our initial assumption that *S. suis* serotype 9 did not contain the *htpsC* gene, our GenBank search revealed that all seven *S. suis* serotype 9 strains with whole genomic sequences do contain the *htpsC* gene, excluding *S. suis* 22,083, whose genomic sequences had not been deposited. It appears that, while most strains of serotype 9 likely contain the *htpsC* sequence, some strains do not. In conclusion, the widespread presence of the *htpsC* sequence among *S. suis* strains indicates the potential of the HtpsC protein as a target antigen for the detection of *S. suis*. This finding could have significant implications for the development of diagnostic tests and potential therapeutics.

Previous studies have demonstrated the utility of colloidal gold-based immunochromatographic assays (GICAs). Yang et al. (2007) developed a GICA to detect antibodies against *S. suis* 2. However, as this assay detects the antibody against the *S. suis* 2 antigen rather than the antigen itself, early infection detection is unlikely. Ju et al. developed a GICA for *S. suis* 2 using colloidal gold-labeled polyclonal antibodies (pAbs), but this method is only suitable for detecting *S. suis* 2 and has a detection limit of 10^6 CFU/mL (Ju et al., 2010). Nakayama et al. designed a GICA for detecting *S. suis* using two prepared pAbs to capsular polysaccharides (CPS) as the capturing antibodies. Although the method shows reasonable specificity and no cross-reaction with control strains such as Streptococcus pneumonia and *Streptococcus lactis*, the results obtained using clinical samples are unstable, with a compliance rate of only 60% (Nakayama et al., 2014).

Compared to these earlier GICA assays, our GICA method for HtpsC detection can identify various *S. suis* strains that express this protein, with a LOD of 1 × 10^3 CFU/mL, showing clear advantages. The test can be completed within 15 min, making it practical for field use. However, further evaluation of our method’s performance with clinical samples is necessary, and the development of additional *S. suis* assays that could be combined with this Immuno-Chromatographic Assay (ICA) might enhance its applicability.

## 4 Conclusion

In this research, we developed a colloidal gold immunochromatographic assay for *S. suis* using a double antibody sandwich design. We used 15 nm colloidal gold to prepare the colloidal gold-polyclonal antibody (pAb) conjugate. The anti-HtpsC-N polyclonal antibody and goat anti-rabbit IgG were immobilized on the nitrocellulose (NC) membrane as the test (T) and control (C) lines, respectively, to assemble the colloidal gold immunochromatographic test strips. We determined that the optimal volume of 1% sodium citrate for generating uniform colloidal gold particles is 2 mL. Additionally, we found that the colloidal gold solution should have a pH of 8.0, and that 6.6 μg of HtpsC pAbs are optimal for preparing gold-labeled antibodies.

Our method can detect multiple serotypes of *S. suis* that contain the HtpsC protein gene. It boasts a detection limit of 1 × 10^3^ CFU/mL, exhibits good specificity, and does not cross-react with several other common pathogenic bacteria, including *E. coli*, *S. enteriditis*, *S. aureus*, *L. monocytogenes*, *S. pyogenes*, *S. agalactiae*, or *E. faecalis*. The method can provide results within 15 min, suggesting promising applications in primary hospitals, remote towns, and outbreak locations.

Furthermore, this research represents the first evaluation of the HtpsC protein as a target antigen for the diagnosis of *S. suis*. Given that the *htpsC* gene is prevalent in multiple serotypes of *S. suis*, the HtpsC protein is likely to be broadly applicable in other diagnostic methods for *S. suis* detection. This opens up new avenues for further advancements in diagnosing *S. suis* infections.

## Data availability statement

The datasets presented in this study can be found in online repositories. The names of the repository/repositories and accession number(s) can be found in the article/Supplementary material.

## Ethics statement

The animal study was approved by Nanjing Bioengineering (Gene) Technology Center for Medicine, Nanjing 210,002, P.R. China. The study was conducted in accordance with the local legislation and institutional requirements.

## Author contributions

YL: Project administration, Writing – original draft. SW: Project administration, Writing – original draft, Data curation. XC: Data curation, Project administration, Writing – original draft, Methodology. MC: Methodology, Conceptualization, Funding acquisition, Resources, Supervision, Validation, Visualization, Writing – review & editing. QL: Project administration, Writing – original draft. DH: Data curation, Writing – original draft, Methodology. QC: Data curation, Writing – original draft. XX: Funding acquisition, Resources, Validation, Writing – review & editing.
