# Supplementary material for: A rapid colloidal gold immunochromatographic assay based on polyclonal antibodies against HtpsC protein for the detection of Streptococcus suis
Source: Front Microbiol. 2023 Nov 21;14:1294368. doi: 10.3389/fmicb.2023.1294368 (PMC10699142; doi:10.3389/fmicb.2023.1294368)
Supplement: Supplementary file 1 [file Data_Sheet_1.docx]

Supplementary material

A rapid colloidal gold immunochromatographic assay based on polyclonal antibodies against HtpsC protein for the detection of *Streptococcus suis*

**Authors:** Yawei Lu^†1^, Sibo Wang^†1^ ,Xushen Cai^†1^, Min Cao*^1^ ,Qingyu Lu^1^, Dan Hu^2^,qiong Chen^2^, Xiaohui Xiong^1^

1. College Food Science and Light Industry, Nanjing Tech University, Nanjing 211816, Jiangsu, P. R. China

2. Nanjing Bioengineering (Gene) Technology Center for Medicine, Nanjing 210002, P.R. China

**Corresponding author: Min Cao*****,** [**anniecao2001@163.com**](mailto:anniecao2001@163.com)**,**

**Co-authors:** Yawei Lu^†1^, Sibo Wang^†1^ ,Xushen Cai^†1^,contributed equally to this work.

| List of Contents: | |
| --- | --- |
| **Figure S1. Expression, purification and identification of recombinant protein HtpsC-N** | P2 |
| **Figure S2. Identification of pAbs by western blot**. | P3 |
| **Figure S3. PCR identification of HtpsC** **gene using various bacteria as template** | P4 |
| **Table S1.** **Identification of HtpsC-N protein by indirect ELISA.** | P5 |
| **Table S2.** **Identification of the sera of rabbits immunized with HtpsC-N protein by indirect ELISA** | P6 |
| **Table S3. Distribution of htpsc sequence in 151 Streptococcus suis strains with deposited genome sequences from Genbank** | P7 |

**C**

**B**

**A**

| 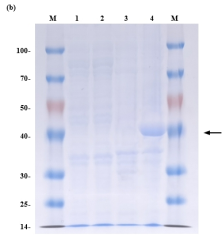 | 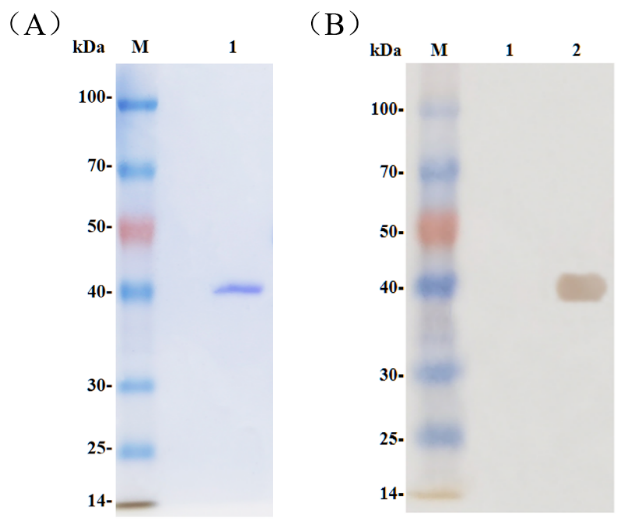 |
| --- | --- |
| **Figure S1. Expression, purification and identification of recombinant protein HtpsC-N** (A) SDS-PAGE analysis of proteins in E. coli before and after induction of the pET-28 expression system. M, Protein Ladder :14~100 kDa. Lane 1, pET-28a control prior to induction. 2. pET-28a control after induction, 3. Recombinant plasmid 28a- HtpsC-N prior to induction, 4. Recombinant plasmid 28a- HtpsC-N after induction. The arrow indicates the recombinant protein in lane 4 migrating at the expected position (41 kDa). (B) SDS-PAGE analysis of HtpsC-N recombinant protein purified by affinity chromatography M, Protein Ladder :14~100 kDa. Lane 1, purified recombinant(C) Western blot identification of protein HtpsC-N after purification by Ni affinity chromatography using s.suis infected pig sera as a probe: M. Protein Ladder 14~100 kDa. Lane 1.pET-28a control ,.Lane 2, purified HtpsC-N recombinant protein identified.  The results showed the HtpsC-N recombinant protein had been successfully expressed and purified and could actively reacted with specific antibodies in s.suis infected pig sera.   \| 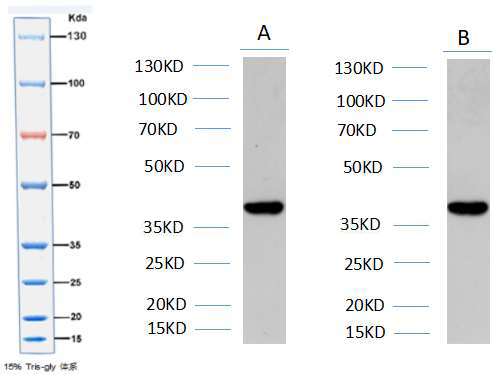 \| \| --- \| \|  \|   **Figure S2 Identification of pAbs by western blot**.  Rabbits were immunized using purified HtpsC-N and boosted as described in Materials and Methods. Serum was collected after the fourth booster. Rabbits were sacrificed and the collected serum was purified by affinity chromatography to obtain anti- HtpsC-N pAbs.. The Western blot shows that the purified anti- HtpsC-N pAbs specifically recognizes HtpsC-N recombinant protein. Lane A and Lane B representative of the western result of anti- HtpsC-N pAbs purified from rabbit A and B respectively.  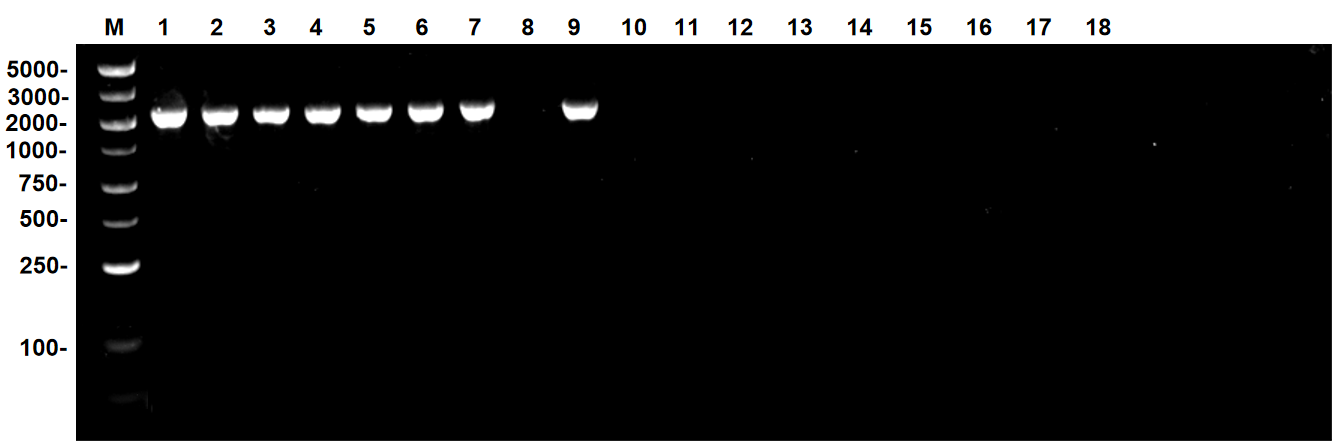  **Figure S3 PCR identification of HtpsC** **gene using various bacteria as template**  M. DNA 分子量标准 5000;Lane 1-9 representative of different types of *S.suis* including *S.suis* 1 5428、*S.suis*2 05ZYH33、*S.suis*2 WZ48-1、*S.suis*2 T15、*S.suis*2 NCTC 10234、*S.suis*4 2524、*S.suis*7 8074、*S.suis*9 22083、*S.suis*14 13730,Lane 10-18 representative of *E.coli* BL21(DE3)、*E.coli* GDMCC 801268、*L. monocytogenes* ATCC 54004、*E. faecalis* ATCC 51299、*S. aureus* ATCC 29213、*S. pyogenes* ATCC 19615、*S. agalactiae* GDMCC 1.408、*S. enteriditis* ATCC 14028、blank control(THB medium) respectively.  Primers for PCR were as the following  Forward primer:AACTTCTTTAGGTTGATTGTTCTC  Backward primer:GATACCCTTCCAGATTCTGATTAT   \| Name \| HtpsC-N \| \| \| \| \| \| Blank control \| \| --- \| --- \| --- \| --- \| --- \| --- \| --- \| --- \| \| Serum \| *S. suis* infected pig sera \| \| \| Normal pig sera \| \| \| \| Number \| 1 \| 2 \| 3 \| 4 \| 5 \| 6 \| \| A450nm \| 0.493 \| 0.550 \| 0.378 \| 0.068 \| 0.060 \| 0.071 \| 0.031 \|   **Table S1**  **Identification of HtpsC-N protein by indirect ELISA.**  HtpsC-N protein was coated in the Microtiter plates (96 well) overnight, *S. suis* infected pig sera and Peroxidase-conjugated goat anti-pig IgG were used as primary and second antibodies respectively, normal pig sera were used as controls in each experiment. The results showed that the OD values of the positive and negative serum samples were significantly different, with OD positive/OD negative >2.1, indicating that the recombinant HtpsC-N protein was active and specifically recognized by the specific antibodies in *S. suis* infected pig sera. | |

| 编号 | 稀释倍数 | A450 nm | |
| --- | --- | --- | --- |
|  |  | Sera of rabbit A | Sera of rabbit B |
| 1 | 1000 | 0.774 | 0.820 |
| 2 | 2000 | 0.728 | 0.839 |
| 3 | 4000 | 0.721 | 0.881 |
| 4 | 8000 | 0.772 | 0.845 |
| 5 | 16000 | 0.792 | 0.803 |
| 6 | 32000 | 0.715 | 0.766 |
| 7 | 64000 | 0.613 | 0.703 |
| 8 | 128000 | 0.609 | 0.685 |
| 0379 | Negative control | 0.041 | 0.038 |
| 10 | Blank control | 0.036 | 0.037 |

**Table S2 Identification of the sera of rabbits immunized with HtpsC-N protein by indirect ELISA**

Serum potency was verified by indirect ELISA assay using recombinant protein as the immunogen. The sera were collected at 128,000-fold dilution for pAbs preparation. The sera of rabbits immunized with BSA were used as negative controls.

| Serial number | Strain name | Genbank ID | Serotypes | Availability of specific sequnece of *Streptococcus suis* | Availability of *htpsc* fragments |
| --- | --- | --- | --- | --- | --- |
| 1 | *Streptococcus suis strain Ssuis_MA8 chromosome, complete genome* | CP085085.1 | 1 | available | exist |
| 2 | *Streptococcus suis strain 2016UMN2965.6 tig00000001, whole genome shotgun sequence* | VIFJ01000001.1 | 1 | available | exist |
| 3 | *Streptococcus suis strain 2015UMN3479.3 tig00000001, whole genome shotgun sequence* | VIFI01000001.1 | 1 | available | exist |
| 4 | *Streptococcus suis strain 2016UMN2965.6 tig00000001_pilon, whole genome shotgun sequence* | VIFD01000001.1 | 1 | available | exist |
| 5 | *Streptococcus suis strain 2017UMN52.2 tig00000001_pilon, whole genome shotgun sequence* | VIFA01000001.1 | 1 | available | exist |
| 6 | *Streptococcus suis strain 2015UMN3479.3 tig00000001_pilon, whole genome shotgun sequence* | VIEY01000001.1 | 1 | available | exist |
| 7 | *Streptococcus suis strain 2016UMN2965.6 tig00000001, whole genome shotgun sequence* | VIET01000001.1 | 1 | available | exist |
| 8 | *Streptococcus suis strain 2017UMN52.2 tig00000001, whole genome shotgun sequence* | VIEQ01000001.1 | 1 | available | exist |
| 9 | *Streptococcus suis strain 2015UMN3479.3 tig00000001, whole genome shotgun sequence* | VIEO01000001.1 | 1 | available | exist |
| 10 | *Streptococcus suis ST1* | CP002651 | 1 | available | exist |
| 11 | *Streptococcus suis JS14* | CP002465 | 1 | available | exist |
| 12 | *Streptococcus suis strain ID35541 chromosome, complete genome* | CP109942 | 1 | available | exist |
| 13 | *Streptococcus suis strain ID38828 chromosome, complete genome* | CP109941 | 1 | available | exist |
| 14 | *Streptococcus suis strain NLS40 chromosome* | CP134477 | 1 | available | exist |
| 15 | *Streptococcus suis strain Ssuis_MA1 chromosome, complete genome* | CP085088 | 1 | available | exist |
| 16 | *Streptococcus suis strain DNR43 chromosome, complete genome* | CP102143.1 | 2 | available | exist |
| 17 | *Streptococcus suis strain DNC49 chromosome, complete genome* | CP102140.1 | 2 | available | exist |
| 18 | *Streptococcus suis strain ISU2660 chromosome, complete genome* | CP031379.1 | 2 | available | exist |
| 19 | *Streptococcus suis strain ISU2414 chromosome, complete genome* | CP030023.1 | 2 | available | exist |
| 20 | *Streptococcus suis strain ISU2514 chromosome, complete genome* | CP030020.1 | 2 | available | exist |
| 21 | *Streptococcus suis strain ISU2614 chromosome, complete genome* | CP031377.1 | 2 | available | exist |
| 22 | *Streptococcus suis strain HA0609 chromosome, complete genome* | CP024126.1 | 2 | available | exist |
| 23 | *Streptococcus suis strain NSUI060, complete genome* | CP012911.1 | 2 | available | exist |
| 24 | *Streptococcus suis strain NSUI002, complete genome* | CP011419.1 | 2 | available | exist |
| 25 | *Streptococcus suis strain TJS75 chromosome, complete genome* | CP095162.1 | 2 | available | exist |
| 26 | *Streptococcus suis strain 2015UMN80.9 tig00000001_pilon, whole genome shotgun sequence* | VIEW01000001.1 | 2 | available | exist |
| 27 | *Streptococcus suis strain 2015UMN80.9 tig00000001, whole genome shotgun sequence* | VIEM01000001.1 | 2 | available | exist |
| 28 | *Streptococcus suis 89-1591 chromosome* | CP095182.1 | 2 | available | exist |
| 29 | *Streptococcus suis 05HAS68* | CP002007 | 2 | available | exist |
| 30 | *Streptococcus suis SC070731* | CP003922 | 2 | available | exist |
| 31 | *Streptococcus suis T15 chromosome* | CP100432 | 2 | available | exist |
| 32 | *Streptococcus suis S735* | CP003736 | 2 | available | exist |
| 33 | *Streptococcus suis A7* | CP002570 | 2 | available | exist |
| 34 | *Streptococcus suis SS12* | CP002640 | 2 | available | exist |
| 35 | *Streptococcus suis GZ1* | CP000837 | 2 | available | exist |
| 36 | *Streptococcus suis 98HAH33* | CP000408 | 2 | available | exist |
| 37 | *Streptococcus suis 05ZYH33, complete genome* | CP000407.1 | 2 | available | exist |
| 38 | *Streptococcus suis strain DE609B chromosome, complete genome* | CP100435 | 2 | available | exist |
| 39 | *Streptococcus suis strain SS15055_N2_C15 chromosome, complete genome* | CP102154 | 2 | available | exist |
| 40 | *Streptococcus suis strain M104300_S20 chromosome, complete genome* | CP102137 | 2 | available | exist |
| 41 | *Streptococcus suis strain STC104 chromosome* | CP100418 | 2 | available | exist |
| 42 | *Streptococcus suis strain STC90 chromosome* | CP100326 | 2 | available | exist |
| 43 | *Streptococcus suis strain STC86 chromosome* | CP100328 | 2 | available | exist |
| 44 | *Streptococcus suis strain STC85 chromosome* | CP100330 | 2 | available | exist |
| 45 | *Streptococcus suis strain STC84 chromosome* | CP100332 | 2 | available | exist |
| 46 | *Streptococcus suis strain STC83 chromosome* | CP100334 | 2 | available | exist |
| 47 | *Streptococcus suis strain STC81 chromosome* | CP100336 | 2 | available | exist |
| 48 | *Streptococcus suis strain STC80 chromosome* | CP100338 | 2 | available | exist |
| 49 | *Streptococcus suis strain STC78 chromosome* | CP100340 | 2 | available | exist |
| 50 | *Streptococcus suis strain LSM178 chromosome* | CP047248 | 2 | available | exist |
| 51 | *Streptococcus suis strain LSM157 chromosome* | CP091422 | 2 | available | exist |
| 52 | *Streptococcus suis strain LSM29 chromosome* | CP091423 | 2 | available | exist |
| 53 | *Streptococcus suis strain SZ1908 chromosome, complete genome* | CP082948 | 2 | available | exist |
| 54 | *Streptococcus suis strain YP20190405 chromosome* | CP065431 | 2 | available | exist |
| 55 | *Streptococcus suis strain 10 chromosome* | CP058742 | 2 | available | exist |
| 56 | *Streptococcus suis strain ISU2714 chromosome* | CP030022 | 2 | available | exist |
| 57 | *Streptococcus suis strain ISU1606 chromosome* | CP030017 | 2 | available | exist |
| 58 | *Streptococcus suis strain CS100322 chromosome, complete genome* | CP024050 | 2 | available | exist |
| 59 | *Streptococcus suis strain SS2-1 chromosome, complete genome* | CP018908 | 2 | available | exist |
| 60 | *Streptococcus suis strain SC19, complete genome* | CP020863 | 2 | available | exist |
| 61 | *Streptococcus suis strain ZY05719, complete genome* | CP007497 | 2 | available | exist |
| 62 | *Streptococcus suis strain Transconjugant cSFJ45 chromosome, complete genome* | CP102748 | 2 | available | exist |
| 63 | *Streptococcus suis strain Transconjugant cAKJ47-2 chromosome, complete genome* | CP102746 | 2 | available | exist |
| 64 | *Streptococcus suis strain TJS56 chromosome, complete genome* | CP095463 | 2 | available | exist |
| 65 | *Streptococcus suis strain Transconjugant cAKJ18 chromosome, complete genome* | CP082201 | 2 | available | exist |
| 66 | *Streptococcus suis strain Transconjugant cDY107 chromosome, complete genome* | CP082200 | 2 | available | exist |
| 67 | *Streptococcus suis strain Transconjugant cSS389 chromosome, complete genome* | CP082197 | 2 | available | exist |
| 68 | *Streptococcus suis strain Transconjugant cFJSM5 chromosome* | CP082199 | 2 | available | exist |
| 69 | *Streptococcus suis strain Transconjugant cNJ3 chromosome* | CP082198 | 2 | available | exist |
| 70 | *Streptococcus suis strain Transconjugant cNJ3 chromosome* | CP082198 | 2 | available | exist |
| 71 | *Streptococcus suis strain SH0104 chromosome* | CP025419 | 2 | available | exist |
| 72 | *Streptococcus suis strain LSM102 chromosome, complete genome* | CP016175 | 2 | available | exist |
| 73 | *Streptococcus suis strain 90-1330 chromosome* | CP012731 | 2 | available | exist |
| 74 | *Streptococcus suis T15 chromosome* | CP100432 | 2 | available | exist |
| 75 | *Streptococcus suis strain INT-01 chromosome, complete genome* | CP041994.1 | 3 | available | exist |
| 76 | *Streptococcus suis strain MY1C3_3B chromosome, complete genome* | CP134487.1 | 3 | available | exist |
| 77 | *Streptococcus suis strain PH2016-081 chromosome, complete genome* | CP134474.1 | 3 | available | exist |
| 78 | *139. Streptococcus suis YB51* | CP078543.1 | 3 | available | exist |
| 79 | *Streptococcus suis ST3* | CP002633 | 3 | available | exist |
| 80 | *Streptococcus suis YB51* | CP078543.1 | 3 | available | exist |
| 81 | *Streptococcus suis YB51* | CP078543.1 | 3 | available | exist |
| 82 | *Streptococcus suis YB51* | CP078543.1 | 3 | available | exist |
| 83 | *Streptococcus suis strain ID34572 chromosome, complete genome* | CP109940.1 | 4 | available | exist |
| 84 | *Streptococcus suis strain ID36054 chromosome, complete genome* | CP109939.1 | 4 | available | exist |
| 85 | *Streptococcus suis strain Ssuis_MA2 chromosome, complete genome* | CP085087.1 | 4 | available | exist |
| 86 | *Streptococcus suis strain Ssuis_MA6 chromosome, complete genome* | CP085086.1 | 4 | available | exist |
| 87 | *Streptococcus suis 6407* | CP008921 | 4 | available | exist |
| 88 | *Streptococcus suis strain TRG6 chromosome* | CP109937 | 4 | available | exist |
| 89 | *Streptococcus suis strain SH1510 chromosome* | CP030124 | 4 | available | exist |
| 90 | *Streptococcus suis strain HA1003 chromosome* | CP030125.1 | 4 | available | absent |
| 91 | *Streptococcus suis strain 1652329 chromosome, complete genome* | CP135093.1 | 5 | available | exist |
| 92 | *Streptococcus suis strain ID48908 chromosome, complete genome* | CP135090.1 | 5 | available | exist |
| 93 | *Streptococcus suis strain 1547095 chromosome, complete genome* | CP135094.1 | 5 | available | exist |
| 94 | *Streptococcus suis strain ID26102 chromosome* | CP135087 | 5 | available | exist |
| 95 | *Streptococcus suis strain ID41570 chromosome, complete genome* | CP079193.1 | 5 | available | absent |
| 96 | *Streptococcus suis strain HN105 chromosome, complete genome* | CP029398.1 | 5 | available | absent |
| 97 | *Streptococcus suis strain 2017UMN1435.22 tig00000001_pilon, whole genome shotgun sequence* | VIFC01000001.1 | 5 | available | absent |
| 98 | *Streptococcus suis strain ID34567 chromosome, complete genome* | CP135089.1 | 5 | available | absent |
| 99 | *Streptococcus suis strain ID24665 chromosome, complete genome* | CP135065.1 | 5 | available | absent |
| 100 | *Streptococcus suis strain ID32563 chromosome, complete genome* | CP135063.1 | 5 | available | absent |
| 101 | *Streptococcus suis strain 3112 chromosome, complete genome* | CP097577.1 | 6 | available | exist |
| 102 | *Streptococcus suis strain 2016UMN1524.93 tig00000001_pilon, whole genome shotgun sequence* | VIFB01000001.1 | 6 | available | exist |
| 103 | *Streptococcus suis strain 1521251 chromosome, complete genome* | CP100431.1 | 7 | available | exist |
| 104 | *Streptococcus suis strain GX69 chromosome, complete genome* | CP071806.1 | 7 | available | exist |
| 105 | *Streptococcus suis strain 13-00283-02 chromosome* | CP058741.1 | 7 | available | exist |
| 106 | *Streptococcus suis strain 2016UMN1524.93 tig00000001, whole genome shotgun sequence* | VIER01000001.1 | 7 | available | exist |
| 107 | *Streptococcus suis D9* | CP002641 | 7 | available | exist |
| 108 | *Streptococcus suis strain SS/UPM/MY/F001 chromosome, complete genome* | CP116393.1 | 8 | available | exist |
| 109 | *Streptococcus suis strain DNR48 chromosome, complete genome* | CP102141.1 | 8 | available | exist |
| 110 | *Streptococcus suis strain WUSS030 chromosome, complete genome* | P110141.1 | 8 | available | exist |
| 111 | *Streptococcus suis strain 2018WUSS151 chromosome, complete genome* | CP101844.1 | 8 | available | exist |
| 112 | *Streptococcus suis strain DNS11 chromosome, complete genome* | CP102152.1 | 9 | available | exist |
| 113 | *Streptococcus suis strain M102942_S11 chromosome, complete genome* | CP102138.1 | 9 | available | exist |
| 114 | *Streptococcus suis strain 16085/3b chromosome* | CP058740.1 | 9 | available | exist |
| 115 | *Streptococcus suis strain GZ0565 chromosome, complete genome* | CP017142.1 | 9 | available | exist |
| 116 | *Streptococcus suis strain NLS50 chromosome, complete genome* | CP134488.1 | 9 | available | exist |
| 117 | *Streptococcus suis D12* | CP002644 | 9 | available | exist |
| 118 | *Streptococcus suis strain DN13 chromosome* | CP015557 | 9 | available | exist |
| 119 | *Streptococcus suis strain 2016UMN125.4 tig00001615_pilon, whole genome shotgun sequence* | VIEX01000007.1 | 10 | available | absent |
| 120 | *Streptococcus suis strain 2016UMN125.4 tig00001615, whole genome shotgun sequence* | VIEN01000007.1 | 10 | available | absent |
| 121 | *Streptococcus suis strain SC183 chromosome, complete genome* | CP071305.1 | 12 | available | exist |
| 122 | *Streptococcus suis strain 2017UMN1435.21 tig00000001_pilon, whole genome shotgun sequence* | VIFE01000001.1 | 12 | available | exist |
| 123 | *Streptococcus suis strain 2017UMN1435.21 tig00000001, whole genome shotgun sequence* | VIEU01000001.1 | 12 | available | exist |
| 124 | *Streptococcus suis strain DNC15 chromosome, complete genome* | CP102148.1 | 16 | available | exist |
| 125 | *Streptococcus suis strain LSS42 chromosome, complete genome* | CP100430.1 | 16 | available | exist |
| 126 | *Streptococcus suis TL13* | CP003993 | 16 | available | exist |
| 127 | *Streptococcus suis strain 684_17B chromosome, complete genome* | CP134489.1 | 16 | available | absent |
| 128 | *Streptococcus suis strain 1522228 chromosome, complete genome* | CP134472.1 | 23 | available | exist |
| 129 | *Streptococcus suis strain ID32098 chromosome, complete genome* | CP082778.1 | 24 | available | absent |
| 130 | *Streptococcus suis strain 39565 chromosome, complete genome* | CP076517.1 | 24 | available | absent |
| 131 | *Streptococcus suis strain ID33329 chromosome, complete genome* | CP068708.1 | 24 | available | absent |
| 132 | *Streptococcus suis strain AH681 chromosome, complete genome* | CP025043.1 | 29 | available | absent |
| 133 | *Streptococcus suis strain M106471_S40 chromosome, complete genome* | CP102135.1 | 30 | available | absent |
| 134 | *Streptococcus suis strain 12RC1 chromosome, complete genome* | CP102094.1 | 31 | available | absent |
| 135 | *Streptococcus suis strain SS389 chromosome, complete genome* | CP082202.1 | 31 | available | absent |
| 136 | *Streptococcus suis strain 2017UMN355.92 tig00000352_pilon, whole genome shotgun sequence* | VIFF01000002.1 | 31 | available | absent |
| 137 | *Streptococcus suis strain 2017UMN1562.911 tig00000001_pilon, whole genome shotgun sequence* | VIEZ01000001.1 | 31 | available | absent |
| 138 | *Streptococcus suis strain 2017UMN355.92 tig00000352, whole genome shotgun sequence* | VIEV01000002.1 | 31 | available | absent |
| 139 | *Streptococcus suis strain 2017UMN1562.911 tig00000001, whole genome shotgun sequence* | VIEP01000001.1 | 31 | available | absent |
| 140 | *Streptococcus suis strain 1081 chromosome* | CP017667.1 | 31 | available | absent |
| 141 | *Streptococcus suis strain 0061 chromosome* | CP017666.1 | 31 | available | absent |
| 142 | *Streptococcus suis strain SRD478 chromosome, complete genome* | CP017088.1 | Nonserotypeable | available | exist |
| 143 | *Streptococcus suis strain M105052_S26 chromosome, complete genome* | CP102136.1 | Nonserotypeable | available | absent |
| 144 | *Streptococcus suis strain TMW_SS028 chromosome, complete genome* | CP134473.1 | Nonserotypeable | available | absent |
| 145 | *Streptococcus suis strain DNS20 chromosome, complete genome* | CP102145.1 | Nonserotypeable | available | absent |
| 146 | *Streptococcus suis strain WUSS351 chromosome, complete genome* | CP039462.1 | Nonserotypeable | available | absent |
| 147 | *Streptococcus suis strain SFJ44 chromosome, complete genome* | CP031970.1 | Nonserotypeable | available | absent |
| 148 | *Streptococcus suis strain 1112S chromosome, complete genome* | CP071697.1 | Nonserotypeable | available | absent |
| 149 | *Streptococcus suis strain 2017UMN355.92 tig00000001, whole genome shotgun sequence* | VIFH01000001.1 | Nonserotypeable | available | absent |
| 150 | *Streptococcus suis strain 2017UMN1435.21 tig00000001, whole genome shotgun sequence* | VIFG01000001.1 | Nonserotypeable | available | absent |
| 151 | *Streptococcus suis strain DNC13 chromosome, complete genome* | CP102149.1 | Nonserotypeable | available | absent |

***Table S3. Distribution of htpsc sequence in 151 Streptococcus suis strains with deposited genome sequences from Genbank***

To investigate the distribution of the *htpsC* gene in *Streptococcus sui*s, we first searched databases using the keyword "*Streptococcus suis*", conditioned by these parameters: Molecule types: genomic DNA/RNA, Source databases: GenBank, and Sequence length: From 1,500,000 to 2,500,000.Our investigation yielded a total of 151 whole genomic sequences of *Streptococcus suis strains* representing various serotypes (1-10, 12, 16, 23, 24, 29, 30, 31 and a minority of non-serotypeable strains) in GenBank. It should be noted that strains of other serotypes might not have been sequenced yet. The *htpsC* gene was found in 119 out of 151 strains (78.8%), with a typical similarity of 99%. No *htpsC* gene was found in the remaining 32 strains. The 119 strains containing the *htpsC* gene were serotyped as 1-9, 12, 14, 16, 23, and a non-serotypeable strain while the 32 strains lacking the *htpsC* gene were serotyped as 4, 5, 10, 16, 24, 29-31, 33 and 9 non-serotypeable strain.
